# Th17 cells sense microbiome to promote depressive-like behaviors

**DOI:** 10.1186/s40168-022-01428-3

**Published:** 2023-04-28

**Authors:** Eva M. Medina-Rodriguez, Jowan Watson, Juliana Reyes, Madhukar Trivedi, Eléonore Beurel

**Affiliations:** 1 Department of Psychiatry and Behavioral Sciences, Miller School of Medicine, University of Miami, Miami, FL 33136 USA; 2 Department of Psychiatry, Center for Depression Research and Clinical Care, University of Texas Southwestern Medical Center, Dallas, TX 75390 USA; 3 Department of Biochemistry and Molecular Biology, Miller School of Medicine, University of Miami, Miami, FL 33136 USA

## Abstract

**Background:**

Microbiome alterations have been associated with depression, and fecal transfer of depressed patients’ microbiomes is sufficient to enhance despair behaviors in rodents. Yet little is known about the potential mechanisms, whereby microbes modulate depressive-like behaviors.

**Results:**

In this study, we showed that certain bacteria known to induce Th17 cells are increased in depressed patients and mice exhibiting learned helplessness. Fecal transfers of human depressed patients’ microbiomes into germ-free-like mice were sufficient to decrease sociability and increased susceptibility to the learned helplessness paradigm, confirming that the microbiome is sufficient to confer depressive-like behaviors. This microbial effect was dependent on the presence of Th17 cells in the recipient, as germ-free-like recipient mice deficient in Th17 cells were resistant to the behavioral changes induced by the microbiome of depressed patients.

**Conclusion:**

Altogether, these findings suggest a crucial role of the microbiome/Th17 cell axis in regulating depressive-like behaviors.

Video Abstract

**Supplementary Information:**

The online version contains supplementary material available at 10.1186/s40168-022-01428-3.

## Introduction

Depression is one of the most prevalent diseases, with a staggering lifetime prevalence of nearly 20% in the USA [1-3]. However, treatment options are quite limited, and many patients fail to have a therapeutic response and/or fail to maintain antidepressant treatment for adequate times [4], emphasizing the great need for improved treatments. Among the potential causative pathways are inflammatory responses, such as cytokine production [5-7], and immune cell production and actions which are regulated by the microbiome [8].

The microbiome is a complex mixture of trillions of microbes (e.g., archaea, bacteria, fungi, protozoa, helminths, and viruses) living in symbiosis with the host [9]. Microbiome alterations in depression have been reported [10-13], such as changes in the relative abundance of Firmicutes, Actinobacteria, and Bacteroidetes compared to healthy individuals [13]. Overall, the profile of the microbiomes in depression is quite different between studies, which might be due to differences in patient antidepressant regimen or diet. Among the consistent findings that microbiome composition is altered in depression are as follows: (i) the decrease of butyrate-producing bacteria (e.g., *Faecalibacterium* and Lachnospiraceae [10, 11]), (ii) the decrease of short-chain fatty acid (SCFA)-producing lactobacilli [14], and (iii) the decrease of Bacteroidetes, which are protective against metabolic diseases [13, 15, 16]. Recently, *Morganella* has been suspected to cause depression [17]. Several mechanistic pathways linking the gut microbiome to the pathophysiology of MDD have been proposed such as modulations of the (i) dopamine system, (ii) production of BDNF, (iii) autonomic nervous system, (iv), stress response, and (v) inflammation [18]. Yet, little is known about the mechanisms, whereby microbes control behaviors.

Transfer of human fecal microbiota from MDD patients to germ-free mice have been used to evaluate the functionality of the microbiome [13, 19-21]. Although transplantation of fecal material is considered a promising treatment option in gastroenteric diseases [22], only 2 cases of MDD patients receiving fecal transplantation as add-on therapy have been reported showing a significant improvement in depressive symptoms after 4 weeks [23]. In rodents, all fecal transfer studies of MDD or HC stools to germ-free rodents assessing behavioral outcomes have focused on changes in immobility in the tail suspension or the forced swim tests, which measure behavioral despair, and have used transfers of pooled donor stools into a large number of germ-free recipient rodents [13, 19-21]. Although these breakthrough reports demonstrated that MDD stools were sufficient to increase behavioral despair in the recipients compared to HC stools, they did not provide evidence for the role of the microbiome in more complex behaviors or the role of the host immune response to the microbiome-induced depressive-like behaviors. In this study, we expanded these findings to other more complex depressive-like behaviors and uncover the role of the host T-helper (Th) Th17 cell response to microbiome changes.

To determine the role of Th17 cells in the recipient mouse after fecal transfer, we used germ-free-like mice depleted in RORγT, the master transcription factor of Th17 cell differentiation [24], or depleted in CCR6, a chemokine receptor that was previously found for Th17 cells to infiltrate the brain [25] and to promote depressive-like behaviors [26] and examine if the microbiome of depressed patients is still able to promote depressive-like behaviors when the recipient mouse Th17 cell response is deficient.

## Materials and methods

### Mice

Six- to 12-week-old wild-type C57BL/6 male mice were used. C57BL/6 mice were bred at the University of Miami Animal Facility. The Rorc (γT)^+/GFP^ mice (strain 007572, [24]) were obtained by crossing Rorc (γT)^+/GFP^ × Rorc (γT)^+/GFP^, to produce 50% Rorc (γT)^+/GFP^, 25% wild type, and 25% Rorc (γT)^GFP/GFP^ mice, and littermates were used. Rorc (γT)^GFP/GFP^ mice were not used because their locomotor activity is altered, compromising the interpretation of the behavioral testing. Mice were housed in light- and temperature-controlled rooms and treated in accordance with NIH and the University of Miami Institutional Animal Care and Use Committee regulations.

### Behavioral assessments

#### Learned helplessness

Learned helplessness was measured using a modified reduced duration inescapable foot shock protocol, as described previously [26, 27]. The reduced duration paradigm was used so wild-type C57BL/6 mice did not develop learned helplessness, allowing measurements of increased susceptibility to learned helplessness. Briefly, mice were placed in one side of a system shuttle box (Med Associates, St. Albans, VT, USA) with the gate between chambers closed. 180 inescapable foot shocks were delivered at an amplitude of 0.3 mA, a duration of 2–6 s per shock, and a randomized inter-shock interval of 5–45 s [26]. Twenty-four hours after the inescapable foot shocks, mice were returned to the shuttle box, and the number of escapes from 30 escape trials was recorded. Each trial uses a 0.3 mA foot shock for a maximum duration of 24 s. The door of the chamber opens at the beginning of the foot shock administration to allow the mouse to escape. Trials in which the mouse did not escape within the 24 s time limit were counted as escape failures. Mice with greater than 15 escape failures were defined as learned helpless [28].

#### Tail suspension test

For the tail suspension test (TST), mice were suspended by the tail on an automated TST cubicle (33 × 31.75 × 33 cm; Med Associates, St. Albans, VT, USA) for a period of 6 min, and the immobile time was analyzed for the last 4 min, using Med Associates software.

#### Open field

The locomotor activity in an open field activity was measured as previously described [27]. Briefly, mice were placed in a Plexiglas open field (San Diego Instrument) outfitted with photobeam detectors under soft overhead lighting, and activity was monitored during 30 min using activity monitoring software (San Diego Instrument).

#### Social interactions

For the three-chambered social interaction test [27], the apparatus was a rectangular, transparent, Plexiglas box divided by Plexiglas walls into three equal-sized connected chambers with an empty wire enclosure in the two end chambers. The day prior to testing, the test mice were habituated individually by being allowed to freely explore the entire apparatus for 20 min, and, separately, an unfamiliar, conspecific, and same-sex stimulus mouse was habituated for 20 min into the wire enclosure in one of the chambers. On the day of the test, the test mouse was placed in the center of the middle chamber and allowed to freely explore the entire apparatus for 5 min. The test mouse was allowed to explore the entire apparatus for 10 min with the unfamiliar mouse placed in one of the chambers on the side of the box. Each session was videotaped and quantified for time spent in each chamber and for number of nose contacts with the stimulus mouse.

### Human samples

Stool samples from 10 participants with current depressive symptoms defined as a Quick Inventory of Depressive Symptomatology (QIDS) score of ≥ 13 and 10 matched healthy controls were analyzed from an ongoing, longitudinal study at the University of Texas Southwestern Medical Center. Samples were collected by participants at home, frozen upon collection, transported frozen back to the center, and then stored at −80 °C until analysis as described in [29]. The study was approved by the University of Texas Southwestern Institutional Review Board. One of the samples of healthy controls was not used for some experiments due to the liquid nature of the sample.

### Shotgun sequencing

#### Extraction of DNA

DNA was isolated using the Quick-DNA™ Fecal/Soil Microbe MiniPrep Kit (Zymo Research), according to the manufacturer’s protocol.

#### Library Prep of DNA

DNA libraries were prepared by CosmosID using the Nextera XT DNA Library Preparation Kit (Illumina) and Nextera Index Kit (Illumina) with total DNA input of 1 ng. Genomic DNA was fragmented using a proportional amount of Illumina Nextera XT fragmentation enzyme. Combinatory dual indexes were added to each sample followed by 12 cycles of PCR to construct libraries. DNA libraries were purified using AMpure magnetic Beads (Beckman Coulter) and eluted in QIAGEN EB buffer. DNA libraries were quantified using Qubit 4 fluorometer and Qubit™ dsDNA HS Assay Kit. Samples were sequenced on an Illumina HiSeq × 2 × 150 bp with a targeted read depth of 3 million total reads per sample.

#### Bioinformatics analysis via CosmosID-Hub

The system utilizes a high-performance data-mining k-mer algorithm that rapidly disambiguates millions of short sequence reads into the discrete genomes engendering the particular sequences. The pipeline has two separable comparators: the first consists of a pre-computation phase for reference databases, and the second is a per-sample computation. The input to the pre-computation phase is databases of reference genomes, virulence markers, and antimicrobial resistance markers that are continuously curated by CosmosID scientists. The output of the pre-computational phase is a phylogeny tree of microbes, together with sets of variable length k-mer fingerprints (biomarkers) uniquely associated with distinct branches and leaves of the tree. The second per-sample computational phase searches the hundreds of millions of short sequence reads, or alternatively contigs from draft de novo assemblies, against the fingerprint sets. This query enables the sensitive yet highly precise detection and taxonomic classification of microbial NGS reads. The resulting statistics are analyzed to return the fine-grain taxonomic and relative abundance estimates for the microbial NGS datasets. To exclude false-positive identifications, the results are filtered using a filtering threshold derived based on internal statistical scores that are determined by analyzing a large number of diverse metagenomes. The same approach is applied to enable the sensitive and accurate detection of genetic markers for virulence and for resistance to antibiotics.

#### LEfSe

Linear discriminant analysis effect size figures were generated using the LEfSe tool from the Huttenhower lab [30], based on relative abundance matrices from CosmosID taxonomic analysis. LEfSe is calculated with a Kruskal-Wallis alpha value of 0.05, a Wilcoxon alpha value of 0.05, and a logarithmic LDA score threshold of 2.0. In the LEfSe figures, red bars to the right convey that the organism in that group is more abundant in the “red” group than the other. Green bars to the left convey that the organism is more abundant in the “green” group. The data were deposited in PRJNA832701.

### Bacterial qPCR

Genomic DNA was purified from stools using the Quick-DNA Fecal/Soil Microbe Miniprep (Zymo Research) according to the manufacturer’s instructions. SFB gene expression (*SFB* primers: 5′-ACGCTACATCGTCTTATCTTCCCGC-3′ and 5′-TCCCCCAAGACCAAGTTCACG-3′, *Clostridium bolteae* 5′-CCTCTTGACCGGCGTGTAACGGCG-3′ and 5′-CTCCACATCACTGTCTTGCTTCC-3′, *Clostridium cf. saccharolyticum* 5′-GATTTGAATGAAGTTTTCGGATG-3′ and 5′-CCTGCACCATGCGGCGCTGTGG-3′, *Clostridium symbiosum* 5′-GAACGAAGCAATTTAACGGAAGT-3′ and 5′-CACACTGTATCATGCGATACTG-3′, *Clostridium hathewayi* 5′- GGTTTCGATGAAGTTTTCGGATG-3′ and 5′-CACCAGACCATGCGGCCCTGTG-3′, *Ruminococcus obeum* 5′-GCACTTGAGCGGATTTCTTCGGA-3′ and 5′-CACACCAGACCATGCGGTCCTG-3′, *Ruminococcus gnavus* 5′-GCGGATTTCTTCGGATTGAAGCA-3′ and 5′-CACACGGTACCATGCGGTACTG-3′, *Butyrate*-producing *bacterium* 5′-GCATTTAGGATTGAAGTTTTCGG-3′ and 5′-CACACTGAATCATGCGATTCTG-3′, *Clostridium* sp. 5′-GATAGTTAGAATGAGAGCTTCGG-3′ and 5′-CTTCCTCAGAAGATGCCTTCCG-3′, *Coprobacillus* sp. 5′-GACGCGAGCACTTGTGCTCGAG-3′ and 5′-CGGTCACCATGCAGTGTCCGTA-3′, *Erysipelotrichaceae bacterium* 5′-GTTTCGAGGAAGCTTGCTTCCAA-3′ and 5′-CTGAGCATGCGCTCTGTATACC-3′, *Subdoligranulum* sp. 5′-GAGGGGAGCTTGCTCCCCAGAGC-3′ and 5′-GATACCAGAATCATGCGGTCCC-3′, *Ruminococcus bromii* 5′-GTTAAGAGAGCTTGCTCTTTTAA-3′ and 5′-GGTCGCTGTACCATGCGATACT-3′, *Firmicutes bacterium* 5′-GGAAATCTCTTCGGAGATGGAAT-3′ and 5′-GACGTTCAAGAGATGCCTCCCA-3′, *Bacteroides dorei* 5′-GGCAGTCAGAGCCATGCGACCC-3′, *Bifidobacterium animalis subsp.lactis* 5′-CCCTGGCAGCTTGCTGCCGGGG-3′ and 5′-CACTCGCATGCGCTCATGTGGA-3′) was assessed by SYBR qPCR in a Jena Analytika instrument, and the results were quantified by the 2^−ΔΔCt^ method. Values were normalized to the total bacteria amount (universal bacterial primers: 5′-ACTCCTACGGGAGGCAGCAGT-3′ and 5′-ATTACCGCGGCTGCTGGC-3′) for each sample.

### Fecal transfer

Specific-pathogen-free (SPF) mice were gavaged with a solution of neomycin (100 mg/kg), metronidazole (100 mg/kg), and vancomycin (50 mg/kg) twice daily for 7 days, and antibiotic treatments were discontinued for 2 days before starting the fecal transfers of HC and depressed patient stools. The behavioral assessments were started a week after colonization. Ampicillin (1 mg/mL) was also provided ad libitum in drinking water. These conditions produced germ-free-like phenotype. Bacterial depletion was evaluated by universal bacterial 16S qPCR. The level of bacteria was 99.9% depleted [31], confirming depletion of the microbiota with the antibiotic regimen. The fecal transfer was achieved by gavage of 100 μL of fecal homogenates of 50 mg of HC or depressed patient stool in PBS, twice in the same day. Fecal colonization after 1 week was confirmed by qPCR.

### Flow cytometry

Immediately after learned helplessness, mice were anesthetized, spleens were recovered, and mice were transcardially perfused with PBS, and brains were removed and processed as previously described [26, 27]. Briefly, the hippocampi were dissected excluding meninges and choroid plexus, passed through a 70 μm cell strainer (BD Bioscience), and the cell suspension was mixed (vol/vol) to obtain a 30% Percoll/R1 medium [RPMI 1640 medium (Corning) supplemented with 1% FBS (Gibco), 100 IU/mL penicillin (Gibco), 100 μg/mL streptomycin (Gibco), 1 × nonessential amino acids (Gibco), 1 μM sodium pyruvate (Gibco), 2.5 μM β-mercaptoethanol (Sigma), and 2 mM L-glutamine (Gibco)]. The cellular suspension was overlaid on 70% Percoll/R1 medium in a centrifuge tube and centrifuged at 2000 rpm for 20 min without using the brake. The cells at the interface of the 30/70% Percoll gradient were recovered, washed once, and resuspended in R10 medium.

For surface staining, cells were stained extracellularly with PerCP-conjugated anti-CD4 (clone RM4-5, eBioscience), BV650-conjugated anti-CD45.2 (clone 104, BioLegend), FITC-conjugated anti-B220 (clone RA3-6B2, eBioscience), PeCy7-conjugated anti-CD11c (clone N418, eBioscience), and PE-conjugated F4/80 (clone BM8, eBioscience). Samples were acquired on a FACSCelesta (BD Bioscience), and data were analyzed with FlowJo software (Tree Star, Inc.).

### *Saa, Il-17a*qRT-PCR

The most distal part of the small intestine was dissected and rinsed, and Peyer’s patches were removed. RNA was extracted with TRIzol reagent (Life Technologies), and cDNA was synthesized with ImProm-II™ Reverse Transcriptase and random primers (Promega). *Saa1*, *Saa2*, *Saa3*, and *Il-17a* expression was measured by SYBR green RT-qPCR in a Jena Analytika instrument, and the results were quantified by the 2^−ΔΔCt^ method. Primers used were the following: *Saa1*: 5′-CATTTGTTCACGAGGCTTTCC-3′ and 5′-GTTTTTCCAGTTAGCTTCCTTCATGT-3′; *Saa2*: 5′-TGTGTATCCCACAAG GTTTCAGA-3′ and 5′-TTATTACCCTCTCCTCCTCAAGCA-3′; *Saa3*: 5′-CGCAGCACGAGCAGGAT-3′ and 5′-CCAGGATCAAGATGCAAAGAATG-3′; and Il-17a: 5′-CTCCAGAAGGCCCTCAGACTAC-3′ and 5′-GGGTCTTCATTGCGGTGG-3′. Values were normalized to *GAPDH* (primers: 5′-AGGTCGGTGTGAACGGATTTG-3′ and 5′-TGTAGACCATGTAGTTGAGGTCA-3′).

### Statistical analysis

Data are represented as mean ± SEM. Outliers were removed if they were 3 standard deviations from the mean. Statistical significance was analyzed with a one- or two-way analysis of variance (ANOVA) for multiple comparisons with Tukey post-hoc test or with Student’s *t*-test or Mann-Whitney using Prism software when appropriate. *p < 0.05 was considered significant. All statistical tests were two sided.

## Results

**Fig. 1:**
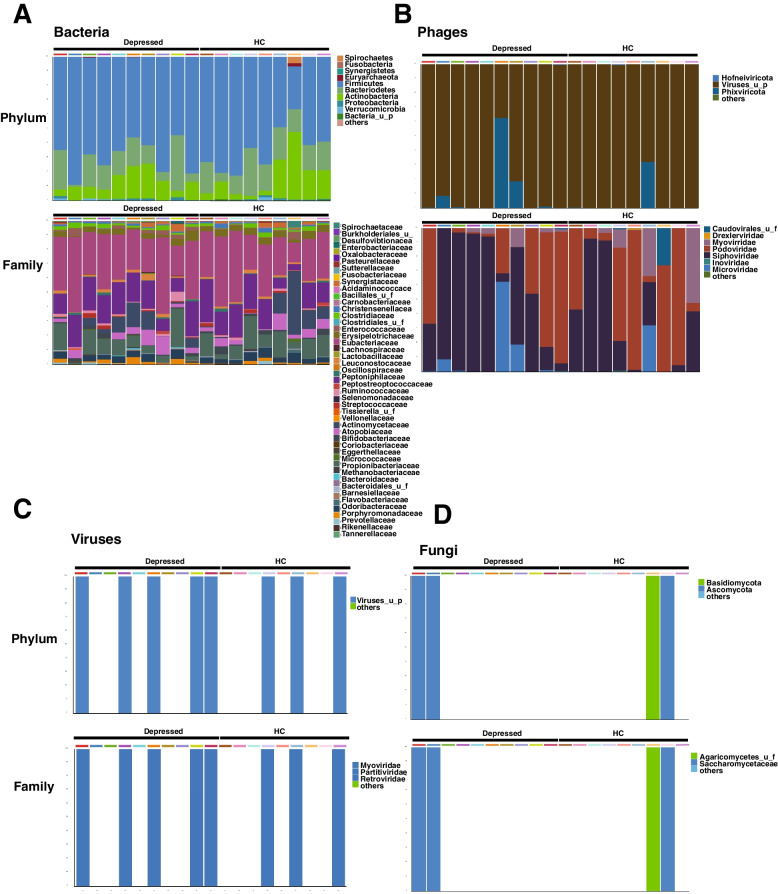
Overview of the abundance and composition of the microbiome of depressed patients and healthy control subjects. Shotgun sequencing was performed with the stools of 10 depressed and 9 matched HC. From read mapping to the genomic database, abundances of bacteria (**A**), phages (**B**), viruses (**C**), and fungi (**D**) were calculated for each microbial taxa across all samples using the CosmosID-HUB. Stacked bar charts show the most abundant phyla (top) and family (bottom) per sample, proportional to the total microbiota within each sample (*N* = 9–10 subjects/group). Charts were generated using normalized, zero-corrected abundance matrices. “Others” represent the abundance data for all other taxa in the abundance data set

**Fig. 2:**
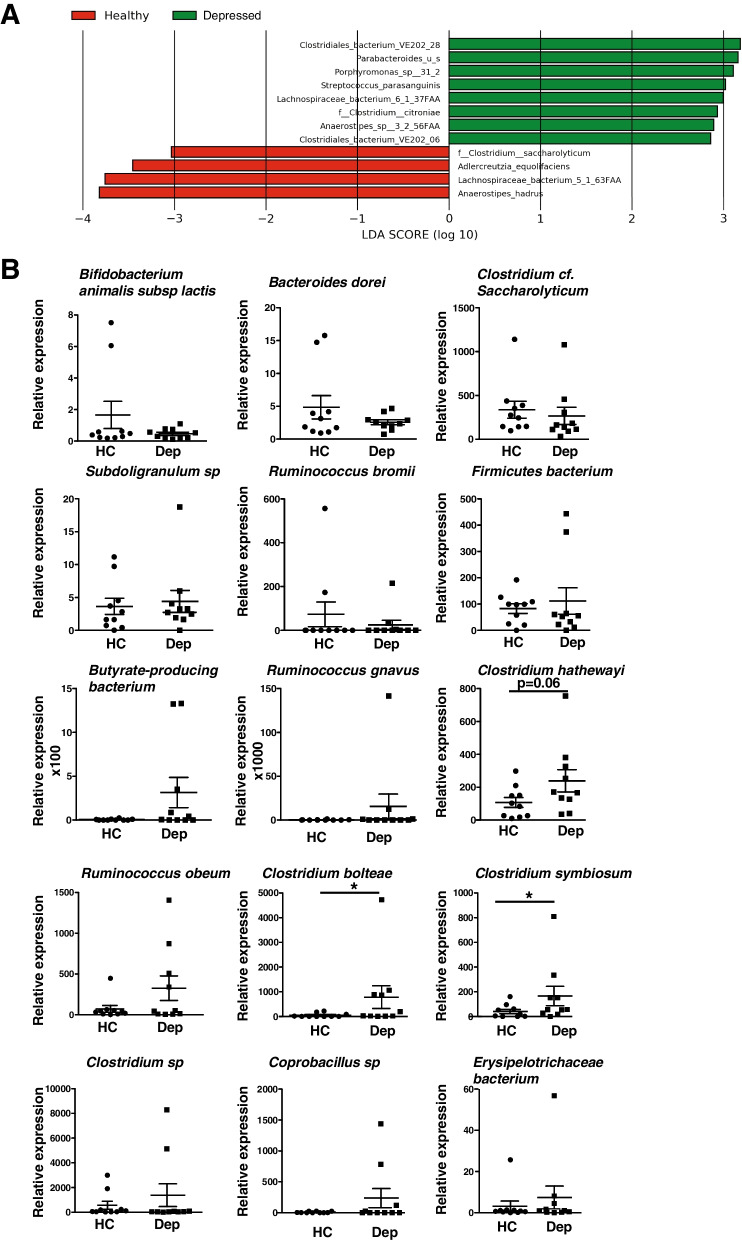
Th17 cell-inducing bacteria are increased in the microbiome of depressed patients compared to healthy controls. **A** LDA is the linear discriminant analysis value from LEfSe and represents the species significantly increased in depressed patients (green) or healthy controls (red) from the analysis of Fig. 1. **B** Fecal relative 16S expression of 15 Th17 cell-inducing bacteria was analyzed by q-PCR. Each symbol represents an individual subject. Data are means ± SEM. *N* = 9–10 subjects/group, Mann Whitney, *U* = 20, *p < 0.05 (*Clostridium bolteae*, *Clostridium symbiosum*), *U* = 25, *p* = 0.0630 (*Clostridium hathewayi*)

We sequenced the gut microbiome of depressed patients and healthy controls by shotgun sequencing and did not find a difference at the Firmicutes and Bacteroidetes phylum or family bacterial levels (Fig. 1A). Furthermore, the presence of viruses or fungi was not different between the depressed patients and the healthy controls (Fig. 1C–D). In contrast, depressed patients appeared to have more Phixviricota at the phage phylum level and more Siphoviridae and Microviridae at the family level (Fig. 1B, Suppl. Fig. 1). When comparing depressed patients and healthy controls at the species bacterial levels, we found that *Clostridiales bacterium*, *Parabacteroides_u_s*, *Porphyromonas* sp., *Streptococcus parasanguinis*, *Clostridium citroniae*, *Lachnospiraceae bacterium*, and *Anaerostipes* sp. are increased in depressed patients (Fig. 2A). Due to the small number of samples, we concentrated on bacteria known to induce Th17 cells [32].

**Fig. 3:**
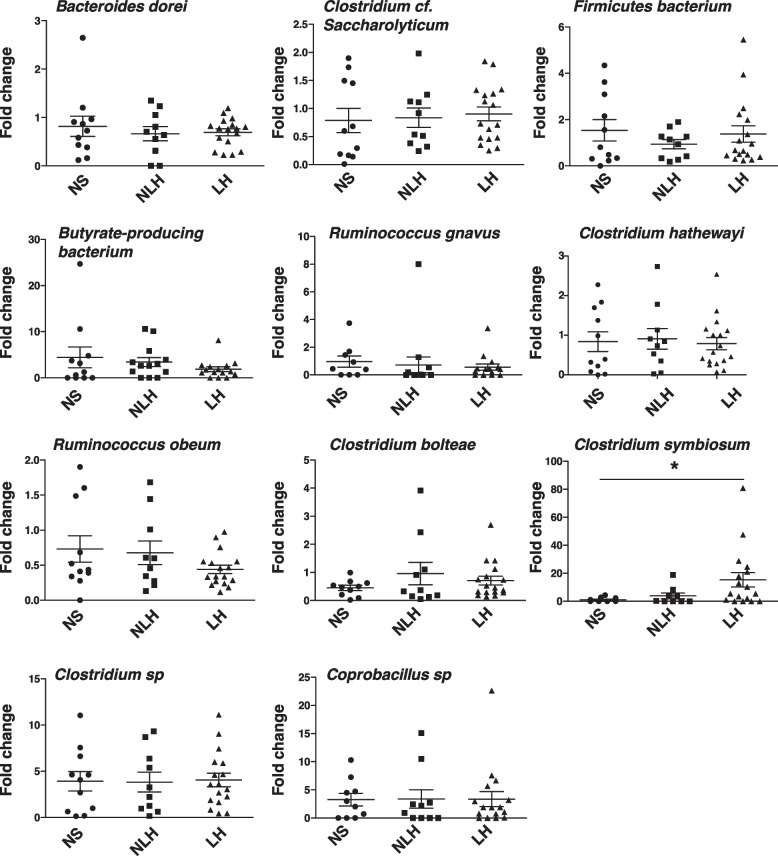
*Clostridium symbiosum* increases in learned helpless mice compared to unstressed mice or non-learned helpless mice. Mice were subjected or not (NS) to the learned helplessness paradigm and divided in 2 groups: learned helpless (LH) (these mice failed to escape > 15/30 trials) and non-learned helpless (NLH) mice. Fecal relative 16S expressions of 11 Th17 cell inducing bacteria were analyzed by q-PCR. Each symbol represents an individual mouse. Data are means ± SEM. *n* = 9–17 mice/group, one-way ANOVA F(2, 33) = 3.334, Tukey post hoc analysis *p < 0.05

**Fig. 4:**
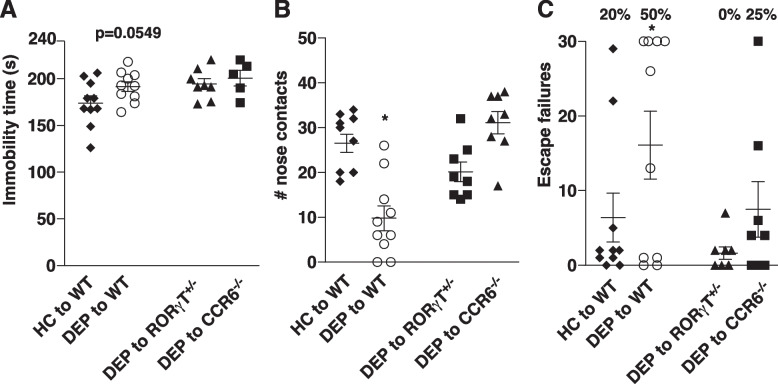
Microbiomes of depressed patients are sufficient to promote depressive-like behaviors in mice and activate a Th17-dependent pathway. Wild-type (WT), RORγT^+/GFP^, CCR6^−/−^ germ-free-like mice received 50 mg of stools resuspended in PBS from healthy controls (HC) or depressed patients (DEP) at a ratio 1 stool for 1 recipient, and immobile time in the tail suspension test (**A**), the number of nose contacts in the sociability test (**B**), and the numbers of escape failures in the learned helplessness paradigm (**C**) were reported. Each symbol represents an individual mouse. Data are means ± SEM. *n* = 5–10 mice/group, one-way ANOVA, F(3, 32) = 2.845, *p* = 0.0549 (TST); F(3, 35) = 15.24 (sociability); F(3, 35) =2.962 (learned helplessness); Bonferroni post hoc analysis *p<0.05

**Fig. 5:**
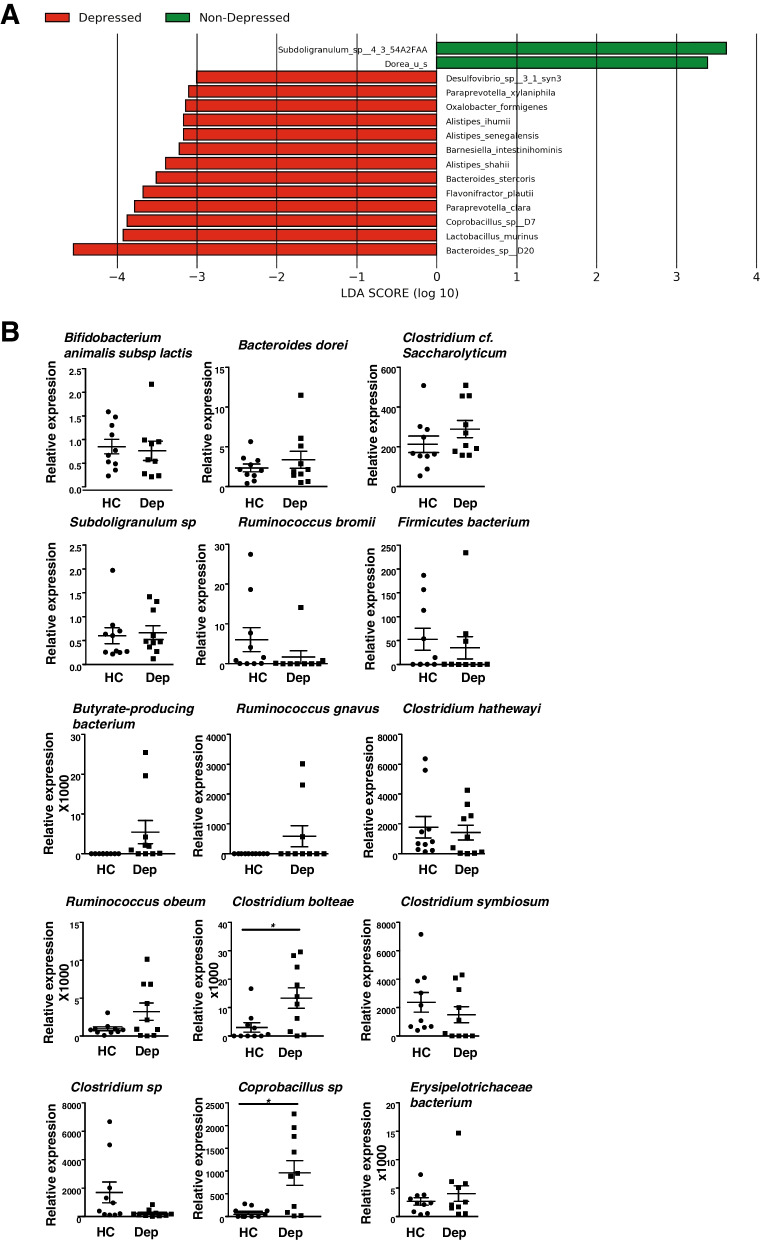
Similar Th17 cell inducing bacteria are increased in the microbiome of mice receiving depressed patients’ microbiomes compared to mice receiving healthy controls’ microbiomes. The microbiome of one subject was transferred to one germ-free-like recipient mouse. And mouse stools were analyzed 10 days after fecal transfer. **A** LDA is the linear discriminant analysis value from LEfSe and represents the species significantly increased in mice receiving depressed patients’ stools and exhibiting learned helplessness (red) or in non-learned helpless mice (green). **B** Fecal relative 16S expression of 15 Th17 cell inducing bacteria was analyzed by q-PCR in the stools of mice receiving either depressed patients’ microbiomes or healthy controls’ microbiomes. Each symbol represents an individual mouse. Data are means ± SEM. *n* = 9–10 mice/group, Mann Whitney, *U* = 18 (*Clostridium bolteae*), *U* = 16 (*Coprobacillus* sp.), *p < 0.05

We analyzed by PCR the levels of bacteria known to promote Th17 cell differentiation (Figs. 2B, 4B, 5). Out of the 15 bacteria measured, we found that 3 bacteria (*Clostridium hathewayi*, *Clostridium bolteae*, *Clostridium symbiosum*) increased in depressed patients compared to matched healthy controls, whereas *Bifidobacterium animalis* subsp. *lactis*, *Bacteroides dorei*, *Clostridium cf. saccharolyticum*, *Subdoligranulum* sp*., Ruminococcus bromii*, and *Firmicutes bacterium* had equivalent levels in the feces of depressed patients and healthy controls (Fig. 2B). When analyzing the levels of some of the same bacteria in mice exhibiting learned helplessness depressive-like behavior, we found that *Clostridium symbiosum* was the only bacteria out of the 11 bacteria measured that was increased in learned helpless mice, whereas other bacteria were unaffected in the feces of learned helpless mice compared to non-learned helpless and to non-shocked mice (Fig. 3). This suggests that *Clostridium symbiosum* might enhance in both human and mice depressive symptoms.

To confirm that the microbiome of depressed patients is sufficient to induce depressive-like behaviors in mice, we used fecal transfer of human stools into C57BL/6 germ-free-like mice and analyzed various depressive-like behaviors. We ensured that the mice were germ-free like by confirming the absence of bacteria [31], and that the mice were colonized 1 week after the human fecal transfer (Suppl. Fig. 2). Consistent with the literature [33-37], fecal transfer of human stools to germ-free-like mice largely recapitulated the human microbiome composition in mice as 100% of phylum, 88% of class, and 84% of genus level taxa were common between the mouse and the human stools, and this was corroborated by no difference in *α* diversity between human and mouse receiving healthy control stools as well as between human depressed patients and healthy controls. However, the species richness of the microbiome of mice receiving fecal transfer of depressed patients was significantly decreased compared to the original human depressed patients’ microbiome (Suppl. Fig. 2A), suggesting that some bacteria from the stools of depressed patients were not able to engraft to the recipient (Suppl. Fig. 2 C–D). Furthermore, the *β*-diversity was different between the human donor and the recipient mouse (Suppl. Fig. 2B), as shown by the variation in the proportion of bacteria between the mouse and human (Suppl. Fig. 2E).

When testing behaviors, we found no difference in the locomotor activity of the mice receiving fecal transfer of depressed patients’ or healthy controls’ stools in an open field (Suppl. Fig. 2F). However, mice receiving fecal transfer of depressed patients’ stool exhibited a slight increase of immobile time in the tail suspension test (Fig. 4A), reduced nose contacts and time spent in the chamber with the novel mouse in the social interaction test (Fig. 4B, Suppl. Fig. 2C), and increased escape failures in the learned helplessness (Fig. 4C) compared to mice receiving fecal transfer of healthy controls’ stools. This confirmed that the microbiota of depressed patients was sufficient to promote depressive-like behaviors of germ-free-like mice. In addition, when comparing bacteria of mice exhibiting depressive-like behaviors to mice that did not exhibit depressive-like behaviors but receiving microbiome of depressed patients, *Coprobacillus* sp. were elevated alongside to other bacteria (Fig. 5A), reinforcing the idea that elevation of bacteria that promote Th17 cells differentiation might be sufficient to promote depressive symptoms. Consistent with this finding, *Clostridium bolteae* and *Coprobacillus* sp. were found elevated in the stools of the mice receiving depressed patients’ stools when compared to mice receiving stools from healthy controls (Fig. 5B).

Because the microbiome shapes the immune system [8], we also examined if fecal transfer of depressed patients’ or healthy controls’ stools affected serum and hippocampal levels of cytokines and splenic and hippocampal percent of immune cells. We found no difference in the levels of 23 hippocampal (Suppl. Fig. 3) and serum (Suppl. Fig. 4) cytokines. This was consistent with no difference in the percent of activated microglia between fecal transfers of depressed patient’s stools and healthy control’s stools (Suppl. Fig. 5A). However, we found a decreased of the percent of hippocampal but not splenic CD4^+^ cells in mice receiving fecal transfer of depressed patient’s stools compared to mice receiving healthy controls’ stools (Suppl. Fig. 5B). Activated macrophages, B cells, and dendritic cells were similar between the 2 groups (Suppl. Fig. 5 C–E).

**Fig. 6:**
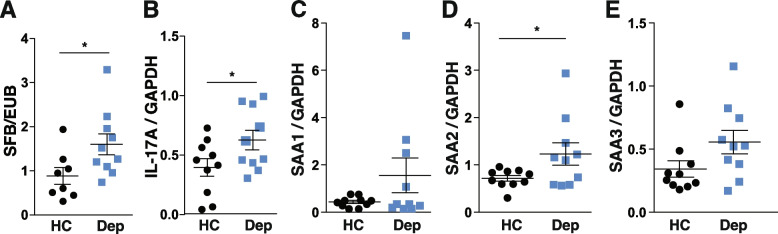
Microbiomes of depressed patients are sufficient to increase SFB/SAA-2/Th17 cell pathway in the intestines of the mice. Fecal SFB (**A**), intestinal IL-17A (**B**), SAA1 (**C**), SAA2 (**D**), and SAA3 (**E**) were analyzed by q-PCR (SFB) and qRT-PCR, respectively (all the others), in mice receiving stools from healthy controls and depressed patients. Each symbol represents an individual mouse. Data are means ± SEM. *n* = 10 mice/group, Mann Whitney, *U* = 14 (SFB); Student *t*-test, *t* = 2.795 (*Il-17a*), *t* = 1.526 (*saa1*) *t* = 2.106 (*saa2*), *t* = 1.898 (saa3), *p < 0.05

Furthermore, we found that mice receiving fecal transfer of depressed patients had increased levels of *Segmented filamentous bacteria (SFB)* (Fig. 6A), of intestinal *Il-17a* (Fig. 6B), and increased levels of serum amyloid A (SAA)2 (Fig. 6D), while they show no difference in the level of SAA1 and SAA3 (Fig. 6 C and E), which corroborated our previous findings that the axis SFB/SAA2/Th17 is activated in depression [31]. It also demonstrated that the microbiome of depressed patients was sufficient to activate this axis in mice.

To determine if the recipient Th17 cells were required to mediate the effects of the microbiome of depressed patients on depressive-like behaviors, we used as recipient, germ-free-like RORγT^+/GFP^ mice (depleted in Th17 cells [24]) and germ-free-like CCR6^−/−^ mice (CCR6 is expressed in some immune cells that migrate to the site of inflammation including Th17 cells [25]). We found that RORγT or CCR6 depletions in recipient mice had no effect on the immobile time in the tail suspension test after fecal transfer of depressed patients’ stools compared to wild-type mice receiving the same stools (Fig. 6A). However, both RORγT and CCR6 in the recipient were required to mediate sociability impairment induced by the fecal transfer of depressed patients’ microbiomes (Fig. 4B and Suppl. Fig. 2G) or to promote susceptibility to the learned helplessness paradigm (Fig. 4C), as RORγT^+/GFP^ and CCR6^−/−^ mice exhibited increased nose contacts and decreased number of escape failures compared to wild-type mice receiving depressed patients’ stools and had similar nose contacts (Fig 4B) and preference for the chamber with the novel mouse (Suppl. Fig. 2G) and number of escape failures as wild-type mice receiving healthy controls’ stools (Fig. 4C). This demonstrated that Th17 cells were required in the recipient to mediate the effects of the microbiota of depressed patients to promote susceptibility to more complex depressive-like behaviors.

## Discussion

In this proof-of-concept study, we report that changes in the microbiome of patients with depressed symptoms are sufficient to promote depressive-like behaviors when transferred to germ-free-like mice, expanding the findings of previous studies on the requirement of the microbiome to promote despair behaviors [13, 19-21] to modulate more complex behaviors such as sociability and learned helplessness, which are two debilitating symptoms associated with depression. Microbiome effect involved the Th17 cell pathway as depletion of Th17 cells in the recipient mice was sufficient to block the effects of depressed patients’ microbiomes. We excluded a massive activation of the immune response both in periphery and in the brain, as blood and hippocampal cytokines were unchanged between mice receiving the microbiome of depressed patients and those receiving the microbiome of healthy controls. However, we found a decrease of hippocampal CD4^+^ T cells, which seems consistent with the findings of immunosuppressed peripheral blood mononuclear cells, in particular reduced levels of blood CD4^+^ T cells in depressed patients [38]. This is consistent with findings that germ-free mice exhibit a more permeable blood brain barrier, and that the blood-brain barrier was restored in germ-free mice receiving fecal transfers [39]. This suggests that microbiome might control CD4^+^ T cell entry to the brain.

It is interesting to note that the microbiota of depressed patients induces social impairments and promotes susceptibility to learned helplessness in addition to despair behaviors, showing the wide impact of the microbiota in controlling depressive-like behaviors. While examining the mechanisms whereby the microbiota controls behaviors, we found that the recipient immune response was required to mediate microbiota effects on sociability and learned helplessness but did not affect the immobile time associated with despair behavior. This is consistent with previous findings that the gut microbiome controls despair behaviors through the host’s metabolism [13] and with our previous findings that the microbiota/Th17 cell axis is important in promoting learned helplessness and in impairing social interactions [31]. In addition, Th17 cells have been implicated in promoting autistic-like traits in mice in a microbiome-dependent manner [32]. Th17 cell induction results from increases of the commensal bacteria SFB in mice [40]). We reported increased SFB level in the depressed patients used in the present study [31]. It is important to note that other bacteria regulating Th17 cell differentiation besides SFB [31] are dysregulated between human and mice exhibiting depressive symptoms, such as *Coprobacillus* sp. and *Clostridium symbiosum*, consistent with previous findings that Coprobacillus and Clostridium are enriched after stress in the gut [41]. In addition, coprobacilli belong to a family of bacteria that increase inflammation [42]. And in the absence of fibers in the diet, coprobacilli damage the protective mucosal layer [43]. Furthermore, *Clostridium bolteae* and *Coprobacillus* sp. have been associated with fast food diet, which is rich in salt [44]. Yet high salt diet affects gut commensal bacteria to induce Th17 cells [45], reinforcing the idea that the diet is likely to impact depressive symptoms, and that changes in nutrition might be beneficial for depressed patients.

It is important to note that there was a change in the richness of the species between the human-depressed donors and the mouse receiving those depressed microbiomes, but this was not observed with the healthy controls’ microbiomes. This suggests that the microbiome of depressed patients might be more difficult to engraft in mice than the microbiome of healthy controls. Although engraftment rules are mainly unknown, it has been proposed that engraftment of human fecal transplantation depends on bacterial abundance, bacterial taxonomy, and elapsed time since the fecal transfer [46]. In our case, it is possible that the mouse environment does not provide the nutrients and adequate conditions necessary for the bacteria enriched in depressed patients’ stools to thrive [47]; whether this is the result of diet variation, genetics, or drug intake remains to be determined. It appears that beneficial bacteria might be the ones failing to engraft, as the microbiomes of depressed patients are still able to induce a Th17 cell response in the recipients reinforcing the idea that the microbiome of depressed patients is less “healthy” and might contribute to depressive symptoms.

Our findings also open new avenues to decipher the role of the microbiome in promoting Th17 cells in human depression. Thus, Th17 cells are increased in the blood of depressed patients [48], and we found increased level of three bacteria known to promote Th17 cell differentiation in depressed patients, and Th17 cells are required to induce the effects of fecal transfer of depressed patients’ microbiome in promoting mouse depressive-like behaviors. Therefore, it is possible that Th17 cells present in the blood of depressed patients are the results of changes in the microbiome, but this will need further testing.

In addition, it is possible that the changes in bacteria associated with depression are dependent on phages. It is important to note that transplantation of the fecal phage fraction alone is sufficient to mediate microbiome effects, suggesting a critical role of phages in modulating physiological processes in the recipients [49, 50]. Little is known about the role of phages in controlling health and disease, but it has been reported that there was a slight increase of the double-stranded DNA phage: Siphoviridae and of the single-stranded DNA phage Microviridae in adults compared to infants, suggesting an accumulation with age of these phages [51]. A decrease of the ratio Microviridae/Caudovirales has been associated with inflammatory bowel disease [52]. Furthermore, Microviridae reside in the genome of Bacteroidetes [53], which are often reduced in depression [54], while Siphoviridae phages seem to have a competitive advantage over other phages to transmit from one species to another. Nevertheless, the role of phages in depression will need further evaluation.

The limitation of this study is the small sample size. Yet, our study is unique because it tackles the functionality of both the donor microbiomes and the immune response of the recipient. Our study had the advantages of analyzing (i) unpooled samples in contrast to previous studies, which pooled samples [13, 19-21], and (ii) the contribution of downstream pathways in the recipient.

In conclusion, the human gut microbiome is sufficient to promote various depressive-like behaviors. This suggests that intervention targeting the microbiome might be beneficial for depressed patients.

## Supplementary Information

**Additional file 1: Suppl. Figure** **1.** Overview of the abundance and composition of the phages in depressed patients and healthy control subjects. Shotgun sequencing was performed with the stools of 10 depressed and 9 matched HC. From read mapping to the genomic database, abundances of phages were calculated for each microbial taxa across all samples using the CosmosID Hub. Stacked bar charts show the most abundant family (A) and species (B) per sample, proportional to the total microbiota within each sample (N= 9-10 subjects/group). Charts were generated using normalized, zero-corrected abundance matrices. **Suppl. Figure 2.** Species richness between human and recipient mouse reconstituted with the human stools. Shannon diversity (A) and Bray-Curtis diversity (B) and heatmap representations of classes (C) and species (D) were analysed in the microbiome of healthy controls (HC), depressed patients (DEP), and mice receiving fecal transfer of HC or DEP stools. E, Comparison at the phylum level of the stools of human subject and of the corresponding recipient mouse 10 days after fecal transfer. Germ-free like wild-type, RORγT^+/GFP^ and CCR6^-/-^ mice received fecal transfer, and a week after colonization, locomotor activity in an open field was assessed (F), and the following day social interaction was evaluated and the time spent in the different chambers (CH1 has the novel mouse, while CH3 does not have a mouse) was recorded (G). Each symbol represents an individual mouse. Data are means±SEM. n=10 mice/group, One-way ANOVA F(3, 37)=6.473 (HC vs.DEP), F(3, 31)=8.431 (CCR6^-/-^ vs. RORγT^+/GFP^), Bonferroni post hoc test *p<0.05. **Suppl. Fig 3.** Hippocampal cytokine levels in mice receiving fecal transfer of depressed patients or healthy controls. Germ-free like mice were gavaged with stools of depressed patients and healthy controls, one week after colonization mice were subjected to behavioral assessments, and sacrificed after the last behavioral test, and cytokines were measured using a multiplex ELISA approach in hippocampal homogenates. Each symbol represents an individual mouse. Data are means±SEM. *n*=7-10/group. **Suppl. Fig** **4.** Serum cytokine levels in mice receiving fecal transfer of depressed patients or healthy controls. Germ-free like mice were gavaged with stools of depressed patients and healthy controls, one week after colonization mice were subjected to behavioral assessments, and sacrificed after the last behavioral test, and cytokines were measured using a multiplex ELISA approach in the serum. Each symbol represents an individual mouse. Data are means±SEM. *n*=7-10/group. **Suppl. Fig 5.** Hippocampal and splenic levels of immune cells in mice receiving fecal transfer of depressed patients or healthy controls. Germ-free like mice were gavaged with stools of depressed patients and healthy controls, one week after colonization mice were subjected to behavioral assessments, sacrificed after the last behavioral test, perfused, hippocampi were recovered and immune cells were analysed by flow cytometry. Activated microglia (F4/80^+^ CD45^int^, A), hippocampal and splenic CD4 cells (B), activated macrophages (F4/80^+^CD45^high^, C), B cells (B220^+^, D) and dendritic cells (CD11c^+^CD45^+^, E) were reported. Each symbol represents an individual mouse. Data are means±SEM. *n* = 9 –10/group. Mann Whitney, U=21, *p=0.0535.

## Data Availability

Data and materials are available upon request.
