# Supplementary figures and images for: Th17 cells sense microbiome to promote depressive-like behaviors
Source: Microbiome. 2023 Apr 28;11:92. doi: 10.1186/s40168-022-01428-3 (PMC10142784; doi:10.1186/s40168-022-01428-3)

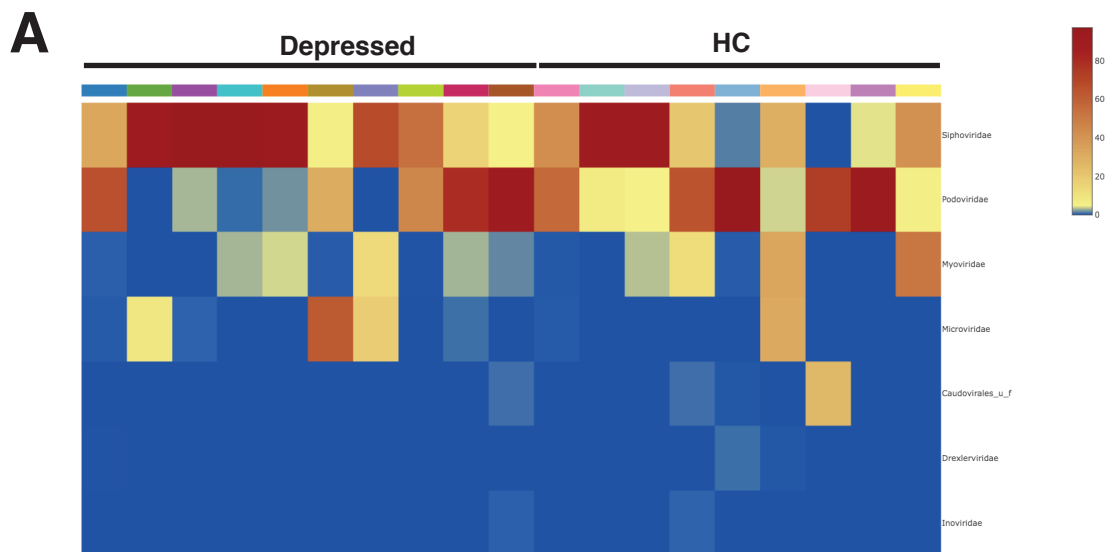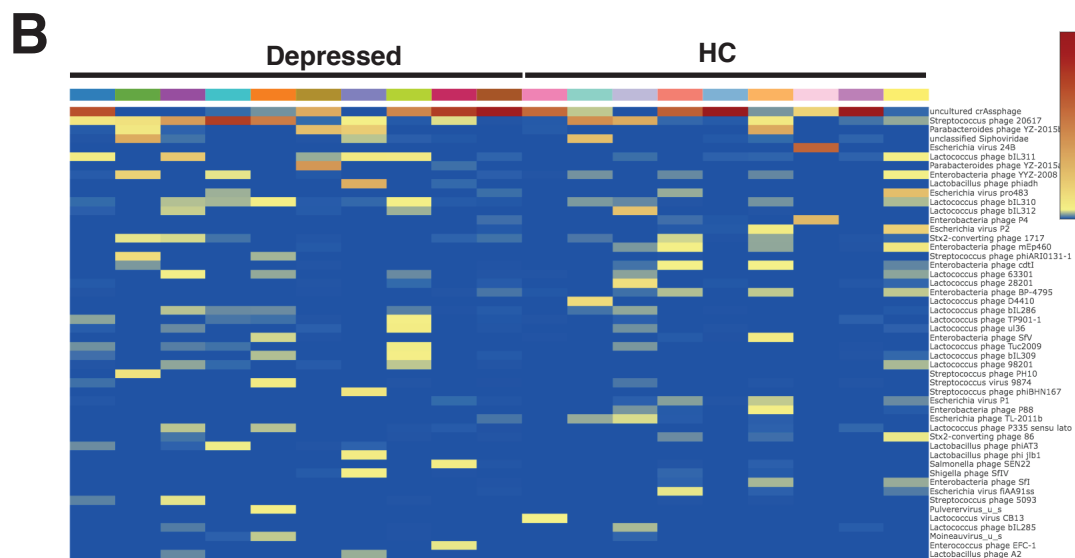

**Suppl. Figure 1**

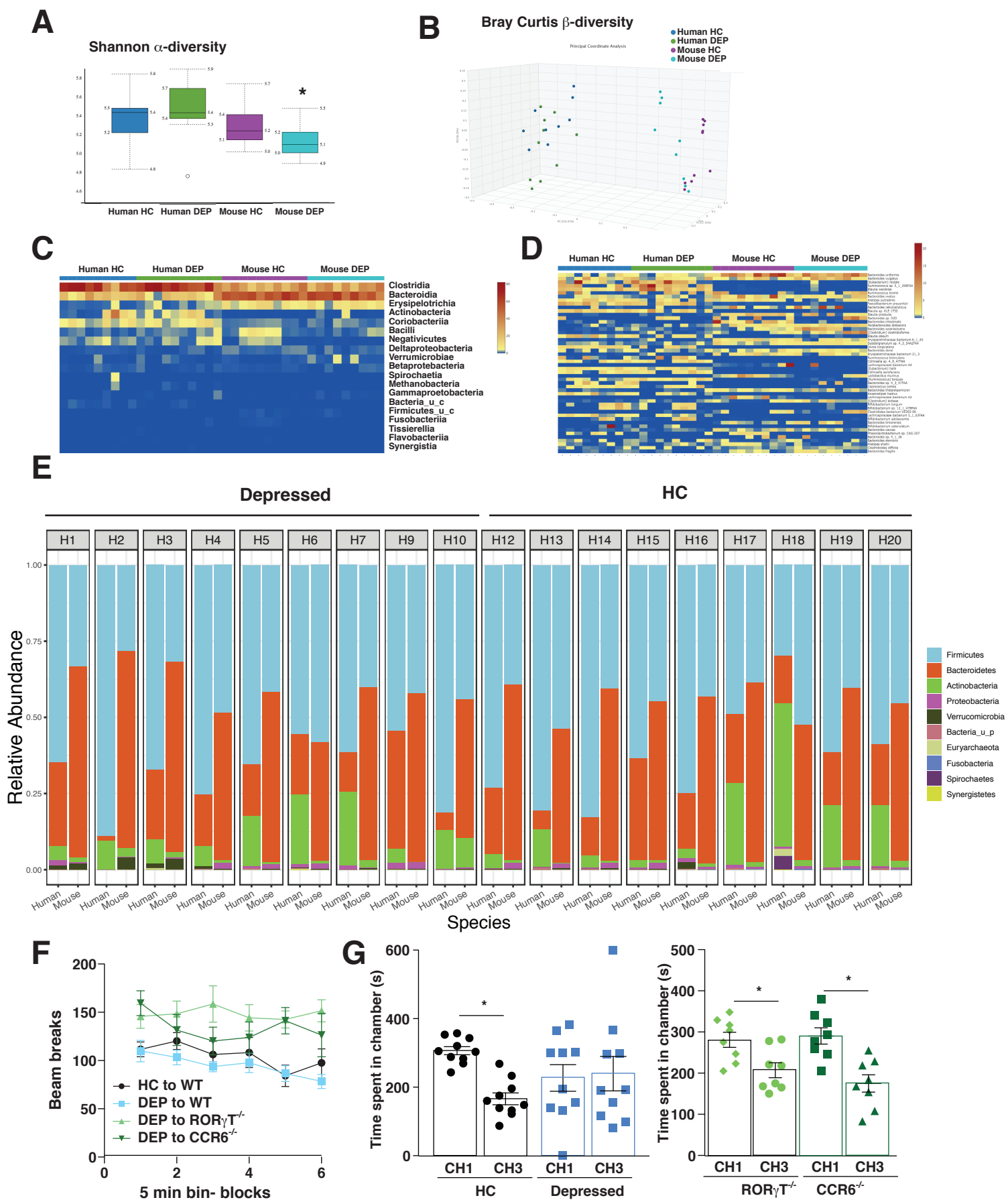

Suppl. Figure 2

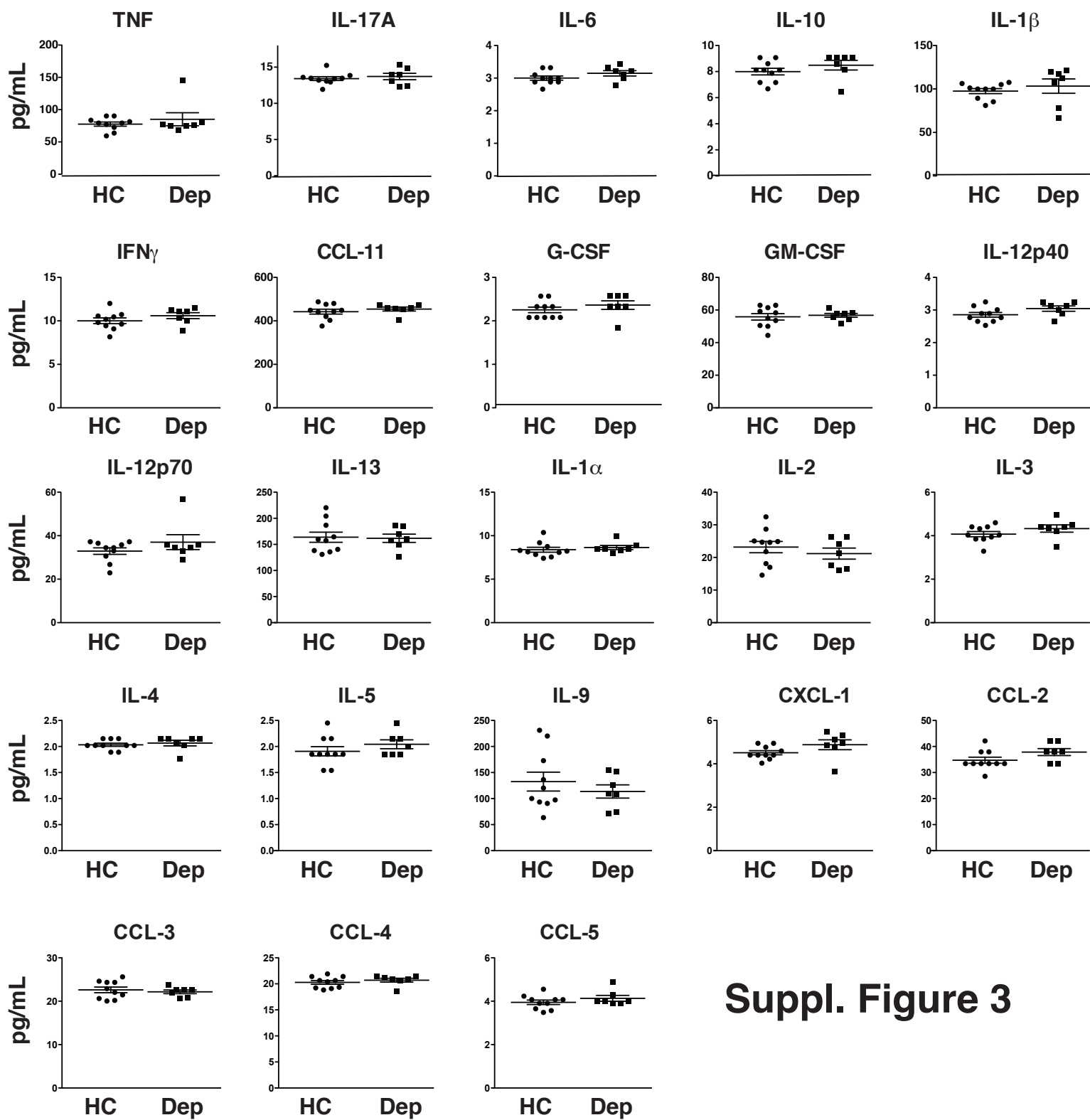

**Suppl. Figure 3**

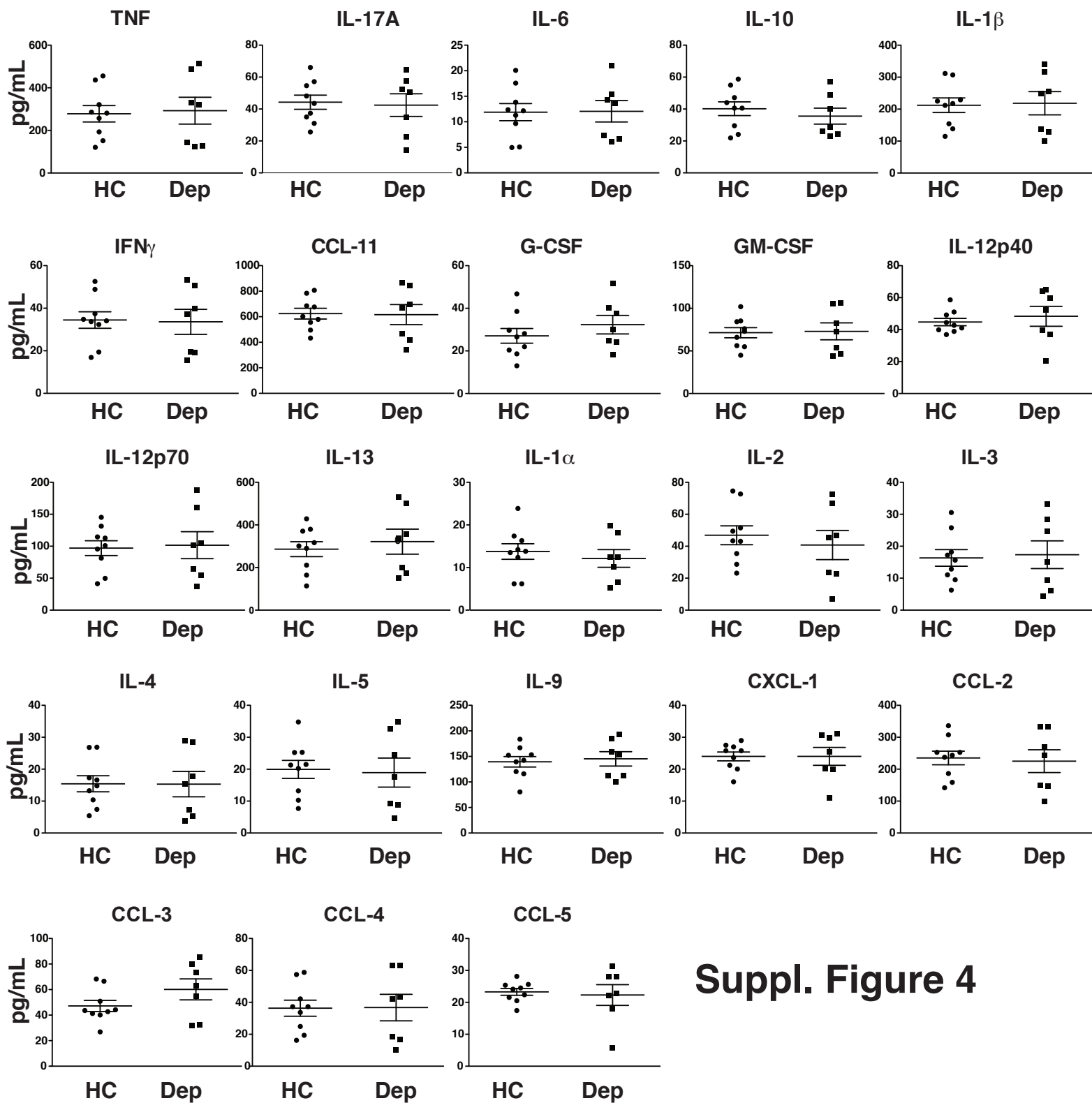

**Suppl. Figure 4**

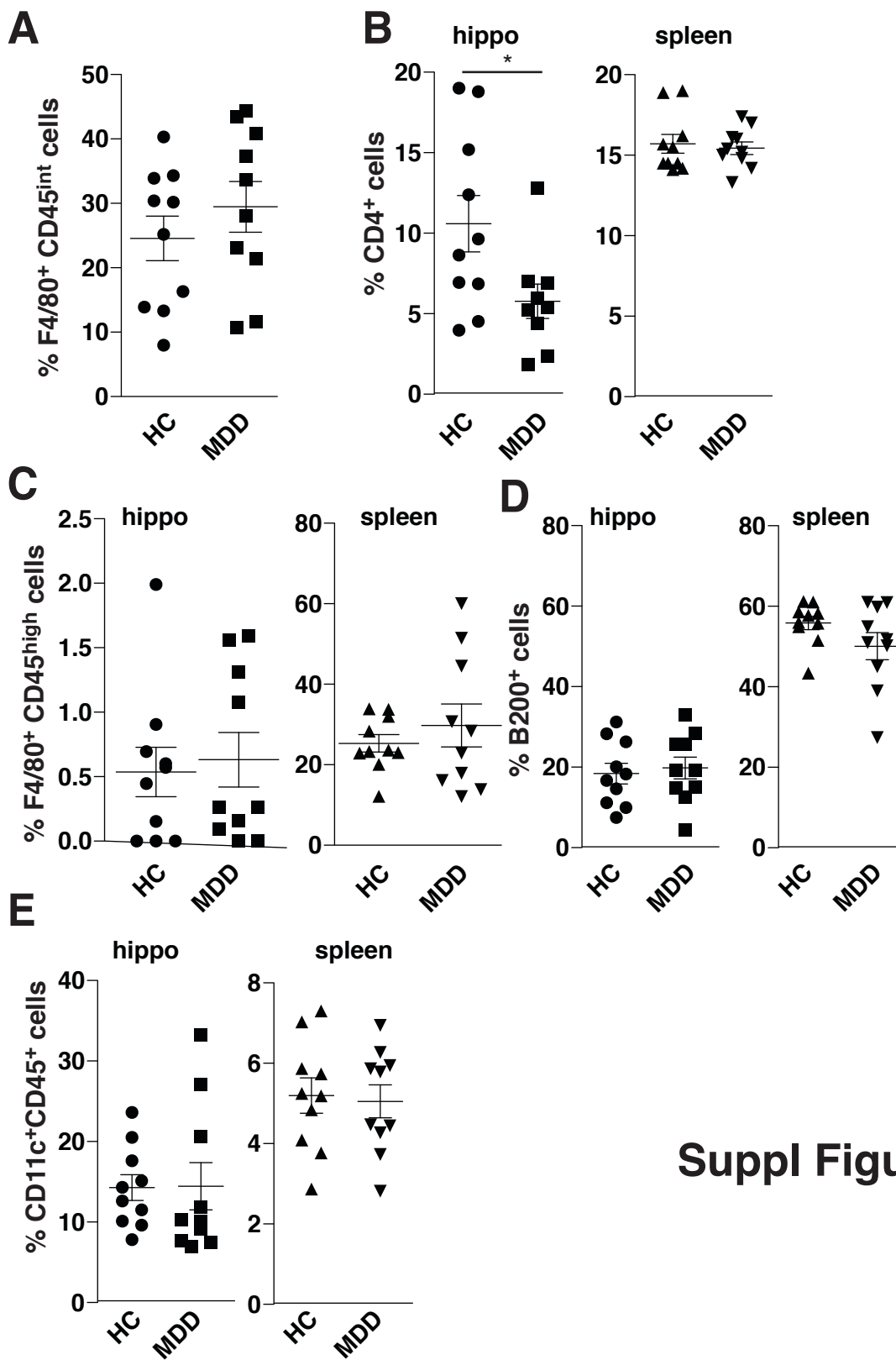

Suppl Figure 5

Supplement: Supplementary file 2 — Additional file 1: Suppl. Figure 1. Overview of the abundance and composition of the phages in depressed patients and healthy control subjects. Shotgun sequencing was performed with the stools of 10 depressed and 9 matched HC. From read mapping to the genomic database, abundances of phages were calculated for each microbial taxa across all samples using the CosmosID Hub. Stacked bar charts show the most abundant family (A) and species (B) per sample, proportional to the total microbiota within each sample (N= 9-10 subjects/group). Charts were generated using normalized, zero-corrected abundance matrices. Suppl. Figure 2. Species richness between human and recipient mouse reconstituted with the human stools. Shannon diversity (A) and Bray-Curtis diversity (B) and heatmap representations of classes (C) and species (D) were analysed in the microbiome of healthy controls (HC), depressed patients (DEP), and mice receiving fecal transfer of HC or DEP stools. E, Comparison at the phylum level of the stools of human subject and of the corresponding recipient mouse 10 days after fecal transfer. Germ-free like wild-type, RORγT+/GFP and CCR6-/- mice received fecal transfer, and a week after colonization, locomotor activity in an open field was assessed (F), and the following day social interaction was evaluated and the time spent in the different chambers (CH1 has the novel mouse, while CH3 does not have a mouse) was recorded (G). Each symbol represents an individual mouse. Data are means±SEM. n=10 mice/group, One-way ANOVA F(3, 37)=6.473 (HC vs.DEP), F(3, 31)=8.431 (CCR6-/- vs. RORγT+/GFP), Bonferroni post hoc test *p<0.05. Suppl. Fig 3. Hippocampal cytokine levels in mice receiving fecal transfer of depressed patients or healthy controls. Germ-free like mice were gavaged with stools of depressed patients and healthy controls, one week after colonization mice were subjected to behavioral assessments, and sacrificed after the last behavioral test, and cytokines were measured u [file 40168_2022_1428_MOESM1_ESM.pdf]
